# Efficacy of *Vitis vinifera* leaf extract and its biosynthesized silver nanoparticles against *Eimeria tenella* in broilers: Effects on performance, anticoccidial indices, and cecal integrity

**DOI:** 10.1016/j.psj.2026.107605

**Published:** 2026-08-12

**Authors:** Hind Alzaylaee, Hend M. Alharbi, Hind Althagafi, Wafa Abdullah I. Al-Megrin, Mohammed M. Qaid, Jameel Al-Tamimi, Saleh Al-Quraishy, Mutee Murshed

**Affiliations:** a Department of Biology, College of Science, Princess Nourah bint Abdulrahman University, P.O. Box 84428, Riyadh, 11671, Saudi Arabia; b Department of Animal Production, College of Food and Agricultural Sciences, King Saud University, P.O. Box 2460, Riyadh 11451, Saudi Arabia; c Department of Zoology, College of Science, King Saud University, P.O. Box 2455, Riyadh 11451, Saudi Arabia

**Keywords:** Broiler performance, *Eimeria tenella*, Intestinal health, Green-synthesized silver nanoparticles, *Vitis vinifera* leaf extract

## Abstract

This study evaluated the anticoccidial efficacy of *Vitis vinifera* leaf extract (VVLE), biosynthesized silver nanoparticles (AgNPs), and their combination (VVLE-AgNPs) in broiler chickens experimentally challenged with *Eimeria tenella*. A total of 150 broiler chicks were randomly assigned to six treatments: infected untreated control (IUC), Tolicox-treated (reference anticoccidial), VVLE, AgNPs, VVLE-AgNPs, and unchallenged untreated control (UUC). Except for the UUC, all birds were orally challenged on day 15 with 1 10^4^ sporulated *Eimeria tenella* oocysts per chick and received their respective oral treatments for 7 days post-inoculation. Parasitological, clinical, growth performance, carcass, and cecal health parameters were evaluated at 7 days post-infection. Infection markedly increased oocyst output, bloody diarrhea, lesion scores, and cecal damage while impairing growth performance in the IUC group (*P* < 0.05). All treatments reduced oocyst output, with VVLE-AgNPs and Tolicox producing the greatest reductions and showing comparable efficacy group (*P* < 0.05). Birds receiving VVLE-AgNPs also exhibited improvements in average daily gain, feed conversion ratio, performance index, and anticoccidial index, approaching values observed in the unchallenged control and exceeding those of VVLE or AgNPs alone group (*P* < 0.05). Carcass yield was not affected (*P* > 0.05). Gross cecal morphometry and histopathological examination demonstrated severe tissue damage in infected untreated birds, whereas VVLE-AgNPs preserved cecal architecture and markedly reduced pathological lesions, with responses comparable to those of Tolicox. In conclusion, VVLE-AgNPs effectively reduced parasite burden, alleviated intestinal damage, and improved productive performance, supporting the potential of plant-mediated AgNPs as a sustainable alternative to conventional anticoccidial drugs for controlling coccidiosis in broilers.

## Highlights

• VVLE-AgNPs significantly reduced oocyst shedding and lesion severity.
• VVLE-AgNPs showed anticoccidial efficacy comparable to the reference drug Tolicox.
• VVLE-AgNPs achieved the highest performance index among all treatments.
• VVLE-AgNPs preserved cecal morphology and reduced histopathological damage.
• The combined treatment outperformed VVLE or AgNPs administered individually.

## Introduction

Coccidiosis is one of the most economically important parasitic diseases affecting the global poultry industry, causing substantial losses through impaired growth performance, reduced feed efficiency, increased mortality, and the costs associated with prevention and treatment (Abdelrahman et al., 2026; Aziz-Aliabadi et al., 2026; Carvalho et al., 2026). Among the seven recognized *Eimeria* species infecting chickens, *Eimeria tenella* (*E. tenella*) is one of the most pathogenic because it primarily invades the ceca, causing severe hemorrhagic enteritis, extensive tissue damage, and impaired nutrient utilization (Al-Baadani et al., 2026; Huang et al., 2018; Kang et al., 2026). Although anticoccidial drugs remain the primary means of disease control, the emergence of drug-resistant *Eimeria* strains, increasing restrictions on chemotherapeutic agents, and consumer demand for residue-free poultry products have intensified the search for safe and sustainable alternatives (Asghar et al., 2026; Debbou-Iouknane et al., 2026; Peek and Landman, 2011). Phytogenic compounds have shown promising anticoccidial activity; however, their application is often limited by poor stability, bioavailability, and inconsistent intestinal delivery. Nanotechnology has therefore emerged as a promising approach to enhance the stability, biological activity, and targeted delivery of phytogenic bioactive compounds.

Phytogenic products are rich in polyphenols, flavonoids, and tannins, and other bioactive metabolites with antimicrobial, antioxidant, anti-inflammatory, and immunomodulatory properties (Aitfella and Aitfella, 2025; Galamatis et al., 2025; Lahlou et al., 2025). Among these, *Vitis vinifera* (grapevine) leaves constitute an abundant source of phenolic compounds and exhibit antioxidant, antimicrobial, and antiparasitic activities (Acero et al., 2025; Murshed et al., 2024; Valencia et al., 2025). Previous studies have demonstrated that plant-derived extracts can interfere with different stages of the *Eimeria* life cycle, reduce oocyst shedding, and improve intestinal integrity in infected birds (Aitfella and Aitfella, 2025; Albarrak and Omar, 2026; Elagib, 2026). In addition to their biological activity, grapevine phytochemicals function as natural reducing and capping agents during plant-mediated nanoparticle synthesis, facilitating the environmentally friendly production of stable metallic nanoparticles without hazardous chemical reagents (Asad et al., 2022; El-Seedi et al., 2019; Kara et al., 2021; Puri et al., 2024).

Recent advances in nanotechnology have expanded the application of plant-mediated AgNPs because of their unique physicochemical properties, high surface-area-to-volume ratio, and broad-spectrum antimicrobial and antiparasitic activities (Abdallah et al., 2025; Barathi et al., 2024; Eker et al., 2025; Frippiat et al., 2025; Gălbău et al., 2026; Hezari et al., 2026; Kakkar et al., 2025; Lasmi et al., 2025; Selvam et al., 2026). In poultry, AgNPs have been reported to improve gut health, modulate immune responses, and reduce pathogen burdens (Al-Sultan et al., 2022; Bolandi et al., 2021; Elbaz et al., 2025). Plant-mediated synthesis further enhances nanoparticle functionality by integrating bioactive phytochemicals with nanomaterials, potentially improving the biological efficacy of phytogenic compounds (Kumar et al., 2024; Madhusudanan et al., 2025; Palomo-Ligas et al., 2023; Pandey et al., 2025; Samanta et al., 2025). Comprehensive physicochemical characterization, including UV-Vis spectroscopy, FTIR, electron microscopy, elemental analysis, and crystallographic techniques, is essential to verify nanoparticle formation and establish relationships between nanoparticle physicochemical properties and biological activity (Asad et al., 2022; El-Seedi et al., 2019; Puri et al., 2024).

Despite the promising antimicrobial and antiparasitic properties of plant extracts and green-synthesized nanoparticles, their combined application as a green nanophytogenic strategy for controlling avian coccidiosis remains largely unexplored. To the best of our knowledge, this is the first *in vivo* study evaluating silver nanoparticles biosynthesized using *Vitis vinifera* leaf extract (VVLE-AgNPs) as an anticoccidial intervention against *E. tenella* infection in broiler chickens. Therefore, the objective of the present study was to evaluate the anticoccidial efficacy of VVLE-AgNPs by assessing growth performance, parasitological indices, anticoccidial index, cecal morphometric responses, gross and histopathological lesions, and overall intestinal integrity in broilers experimentally challenged with *E. tenella*.

## Materials and methods

### Ethical approval

All experimental procedures involving animals were approved by the King Saud University (KSU) Scientific Research Ethics Committee (Approval No. KSU-SE-25-10) following review by the Departmental Board of Studies on Ethics, Methodology, and Animal Welfare. The study was conducted in accordance with institutional guidelines for the care and use of animals and complied with the ARRIVE 2.0 guidelines.

### Experimental design, birds, management, diets, and treatments

A total of 150 one-day-old Ross 308 broiler chicks (initial body weight, 43.85 ± 0.40 g) were randomly allocated to six treatment groups in a completely randomized design, with five replicate cages per treatment and five chicks per replicate (*n* = 25 birds per treatment). Birds were reared from 1 to 21 days of age in a climate-controlled facility at the Department of Zoology, KSU, Riyadh, Saudi Arabia.

Chicks were housed in galvanized wire battery cages (50 × 50 × 30 cm), providing 0.25 m^2^ of floor space per replicate (0.05 m^2^ per bird). Ambient temperature was kept at 35°C on the 1rs^t^ day and gradually reduced to 24°C by 21 days of age. A continuous lighting program (24 h light) was provided during the first week, followed by a 23 h light:1 h dark schedule thereafter. Feed and water were offered *ad libitum* throughout the experiment. Chicks were vaccinated at the hatchery against infectious bronchitis (IB), infectious bursal disease (IBD), and Newcastle disease (ND) according to standard commercial vaccination practices.

Corn-soybean meal diets were formulated to meet or exceed the nutrient requirements recommended for Ross 308 broilers during the starter (0-14 d) and grower (15-21 d) phases and were offered in mash form. The ingredient composition and calculated nutrient contents of the diets are presented in Supplementary Table S1.

The experimental treatments consisted of: (1) infected untreated control (IUC), (2) Tolicox-treated group, (3) VVLE, (4) AgNPs, (5) VVLE-AgNPs combination, and (6) unchallenged untreated control (UUC).

### Preparation of VVLE and AgNPs

Fresh VVLEs were collected from wild populations in Riyadh, Saudi Arabia (24°24′N, 46°43′E). Voucher specimens were authenticated and deposited in the Herbarium of the Department of Botany and Microbiology, King Saud University. Leaf extraction was performed according to Murshed et al. (2024). Briefly, fresh VVLEs were washed, air-dried at room temperature, and ground into a fine powder. Ten grams of leaf powder were extracted with 400 mL methanol (25 mg/mL) under continuous shaking for 24 h at room temperature. The extract was filtered through Whatman No. 1 filter paper and concentrated under reduced pressure at 40°C using a rotary evaporator (Yamato RE300, Tokyo, Japan). The concentrated extract was dried and stored for subsequent analyses. A portion of the filtrate was used immediately for nanoparticle synthesis, whereas the remaining extract was evaporated to dryness, reconstituted in distilled water, and stored at −20°C until use.

### AgNPs biosynthesis and characterization

AgNPs were synthesized following the method of Anandalakshmi et al. (2016). Briefly, 5 mL of VVLE was mixed with 45 mL of 8 × 10^-3^ M silver nitrate solution and incubated at 50°C for 60 min. The formation of VVLE-AgNPs was initially indicated by a color change of the reaction mixture from light yellow to dark brown and subsequently confirmed by ultraviolet-visible (UV-Vis) spectroscopy through detection of the characteristic surface plasmon resonance absorption peak. The physicochemical properties of the synthesized nanoparticles were further characterized by Fourier-transform infrared (FTIR) spectroscopy to identify the functional groups involved in nanoparticle reduction and stabilization, transmission electron microscopy (TEM) to examine nanoparticle morphology and particle size, and energy-dispersive X-ray spectroscopy (EDX) to determine elemental composition. The crystalline structure of the biosynthesized nanoparticles was verified by X-ray diffraction (XRD) analysis (Asad et al., 2022).

### Preparation of E. tenella inoculum and experimental challenge

Unsporulated *E. tenella* oocysts were isolated from naturally infected chickens and allowed to sporulate in 2.5% potassium dichromate solution at 25°C for 72 h. The sporulated oocysts were purified using a salt flotation technique and stored at 4°C until use, following the method described by Takano et al. (2025). Species identification was confirmed based on oocyst morphology, characteristic cecal lesions, and molecular analysis of the internal transcribed spacer 1 (ITS-1) region performed by researchers at the Parasitology Laboratory, Department of Zoology, KSU. To preserve infectivity, the oocysts were propagated through two to three successive passages in coccidia-free broiler chickens. A preliminary challenge trial was conducted to establish an inoculation dose that consistently induced cecal lesions while minimizing mortality.

At 14 days of age, all chicks except those in the UUC group were orally challenged with 1 × 10⁴ sporulated *E. tenella* oocysts/ 100 μL DW/ chick. The UUC group received distilled water only. Two hours after challenge, treatments were initiated and administered orally. Chicks in the VVLE group received 100 mg/kg body weight of VVLE, whereas those in the AgNPs group received 50 mg/kg body weight of AgNPs. Chicks in the VVLE-AgNPs group received a combination of 50 mg/kg body weight VVLE and 50 mg/kg body weight AgNPs. These doses were selected based on preliminary dose-response studies.

The reference anticoccidial treatment consisted of toltrazuril (Tolicox®), administered via drinking water at 7 mg/kg body weight daily for four consecutive days according to the manufacturer's recommendations and previous studies evaluating anticoccidial efficacy (Na et al., 2026). The VVLE, AgNPs, and VVLE-AgNPs treatments were administered daily for seven days post-infection.

### Performance indices

Body weight and feed intake were recorded on a replicate basis throughout the experimental period. Average daily gain (ADG), average daily feed intake (ADFI), and feed conversion ratio (FCR) were calculated, with FCR determined as the ratio of ADFI to ADG. Performance index (PI) was calculated using the following equation:

$$\text{PI}=\frac{\text{Live}\;\text{body}\;\text{weight}\;\left(\text{kg}\right)}{Feed\;conversion\;ratio}\times \;100.$$

### Carcass traits, cecal morphometry, and cecal lesion score

At 7 DPI, one broiler was randomly selected from each replicate cage (*n* = 5 per treatment) for carcass and organ evaluation. Broilers were fasted for 8 h, individually weighed, and humanely slaughtered by severing the jugular vein in accordance with halal standards and the ARRIVE 2.0 guidelines. Following defeathering and evisceration, carcass weight was recorded immediately, and carcass yield was calculated according to Islam et al. (2026) as:

$$\frac{\text{Carcass}\;\text{weight}\;\left(g\right)}{\text{Pre}\;\text{slaughter}\;\text{weight}\;\left(g\right)}\times \;100$$

Breast muscle, leg yield, spleen, bursa of Fabricius, thymus, pancreas, and edible giblets (liver, heart, and gizzard) were weighed individually and expressed as a percentage of carcass weight using the following equation.

$\frac{Carcass\;part\;weight\;(g)}{\text{Carcass}\;\text{weight}\;(g)}\times \;100$, as described by Nowaczewski et al. (2026).

Cecal dimensions were achieved on each slaughtered broiler and included absolute cecal weight, cecal length, and cecal wall thickness as described by Nerida et al. (2024). Cecal length (cm) was measured using a ruler as a gross indicator of cecal atrophy, while cecal weight (g) was recorded with a digital electronic weighing balance. Cecal wall thickness was expressed as the cecal weight: length ratio (g/cm), which was used as a practical gross anatomical indicator of cecal tissue hypertrophy and inflammatory changes as previously described by Qaid et al. (2026). This ratio provides a standardized measure that integrates cecal mass relative to its length and was therefore used to facilitate comparisons among treatment groups. However, it should be considered an indirect measure of wall thickness, and direct histological measurements of cecal wall thickness would provide greater anatomical precision.

### Clinical signs score, fecal bloody diarrhea, lesion score, and fecal oocyst count

Clinical signs of coccidiosis, including depression, reduced feed intake, ruffled feathers, drooping wings, decreased activity, diarrhea, and bloody feces, were assessed daily after challenge. Severity was scored on a 0-4 scale (0 = absent, 4 = severe), and the highest score observed per replicate was used for statistical analysis. Bloody diarrhea was evaluated daily from 4 to 7 DPI using a 0-4 scoring system (0 = normal feces; 4 = severe bloody diarrhea), and the highest score recorded per replicate was used for analysis. The percentage reduction in bloody diarrhea relative to the IUC group was calculated according Galamatis et al. (2025):

$$\frac{\text{Highest}\;\text{score}\;\text{in}\;\text{IUC}\;-\text{Highest}\;\text{score}\;\text{in}\;\text{treated}\;\text{group}}{\text{Highest}\;\text{score}\;\text{in}\;\text{IUC}\;\text{group}}\times \;100.$$

At 7 DPI, excreta samples were collected from each replicate, homogenized, and suspended in 10 mL of water. Oocyst output per gram of feces (OPG) was determined using the McMaster counting procedure as described by Qaid et al. (2021b). The oocyst inhibition rate (%) was calculated as an indicator of anticoccidial efficacy (Al-Baadani et al., 2026).

$$\frac{\text{Mean}\;\text{OPG}\;\text{in}\;\text{IUC}-\;\text{Mean}\;\text{OPG}\;\text{in}\;\text{treated}\;\text{group}}{\text{Mean}\;\text{OPG}\;\text{in}\;\text{IUC}}\times 100$$

At necropsy (7 DPI), cecal lesions were scored macroscopically according to Chen et al. (2025) using a 0-4 scale, where 0 = normal ceca and 4 = severe hemorrhage with darkened ceca and possible mortality.

### Evaluation of positive and negative anticoccidial indicators

Relative body weight gain (RBWG, %) and survival rate (%) as positive indicators and oocyst value (%) and lesion score as negative indicators used for calculating the anticoccidial index (ACI). Survival rate (%) was calculated as the proportion of surviving broilers relative to the initial number of broilers in each treatment group as following equation:

ACI=(RelativeBWG+Survivalrate)−(Oocystvalue+Lesionscore)

The ACI was interpreted as follows: <120 = inactive, 120-140 = slight, 140-160 = moderate, 160-180 = marked, and >180 = excellent anticoccidial activity (Lan et al., 2016).

Relative oocyst production (oocyst value, %) was calculated as follows (Llalla Vidal et al., 2025):

$$Oocyst\;value\;\left(\%\right)=\frac{\text{Mean}\;\text{OPG}\;\text{in}\;\text{treated}\;\text{infected}\;\text{group}}{\text{Mean}\;\text{OPG}\;\text{in}\;\text{treated}\;\text{IUC}\;\text{group}}\times \;100$$

RBWG (%) was determined by comparing the daily gain (ADG) of each treatment with that of the UUC group as:

$$\text{RBWG}\;\left(\%\right)=\frac{\text{ADG}\;of\;treated\;group}{\text{ADG}\;\text{of}\;\text{UUC}\;\text{group}\;}\times \;100$$

Survival rate (%) was calculated as:

$$\text{Survival}\;\text{rate}\;\left(\%\right)=\frac{Number\;of\;surviving\;birds}{\text{Initial}\;\text{number}\;\text{of}\;\text{birds}}\times \;100$$

These lesion scores were subsequently incorporated into the ACI calculation.

### Cecal histopathology

At 7 DPI, approximately 2 cm of cecal tissue samples were collected from one broiler per replicate, rinsed with phosphate-buffered saline (PBS), and fixed in 10% neutral-buffered formalin for 48 h. Samples were dehydrated through a graded ethanol series, cleared in xylene, embedded in paraffin wax, sectioned at 5 μm thickness using a rotary microtome, and stained with hematoxylin and eosin (H&E) (Al-Baadani et al., 2026). Histological sections were examined under a light microscope (Olympus, Tokyo, Japan) at 100x magnification to evaluate cecal mucosal architecture, inflammatory cell infiltration, epithelial degeneration, hemorrhagic lesions, and the presence of endogenous developmental stages of *E. tenella*. Representative photomicrographs were obtained from each treatment group for qualitative histopathological assessment and comparison.

### Statistical analysis

Data were analyzed using SAS (2012) under a completely randomized design included treatment as a fixed effect. A one-way analysis of variance (ANOVA) was conducted using the General Linear Model (GLM) procedure. The statistical model was expressed as follows:

$$\gamma_{ij}=\mu +T_{i}\;+e_{ij}$$

Where *Y_ij_* denotes the individual observation, μ is the overall mean, *T_i_* denotes the effect of the i^th^ treatment, and *e*_ij_ is the random residual error.

For growth performance variables, the pen (replicate) was considered the experimental unit, whereas for carcass characteristics, cecal morphometric measurements, and histopathological evaluations, one bird randomly selected from each replicate served as the experimental unit (*n* = 5 per treatment) consistent with the sampling approach used in previous studies (Qaid et al., 2026, 2021c). Prior to analysis, data were assessed for normality (Shapiro–Wilk test) and homogeneity of variance (Levene's test). No significant deviations from normality or homogeneity of variance were detected (*P* > 0.05), indicating that the data met the assumptions required for parametric analysis. When treatment effects were significant, means were separated using Tukey's multiple comparison test. Differences were considered statistically significant at *P* < 0.05.

## Results

### Characterization of AgNPs

UV-Vis spectroscopy confirmed the successful biosynthesis of silver nanoparticles through the appearance of the characteristic surface plasmon resonance absorption peak, indicating the reduction of Ag⁺ ions into metallic silver nanoparticles (Supplementary Fig. S1). FTIR analysis demonstrated the presence of functional groups derived from *Vitis vinifera* leaf phytochemicals that likely participated in nanoparticle reduction and surface stabilization (Supplementary Fig. S2A). TEM examination revealed predominantly well-dispersed nanoparticles with nanoscale dimensions and approximately spherical morphology (Supplementary Fig. S2B). EDX analysis confirmed silver as the principal elemental constituent together with minor signals corresponding to plant-derived elements involved in nanoparticle capping (Supplementary Fig. S2C). Furthermore, XRD analysis demonstrated distinct diffraction peaks corresponding to crystalline metallic silver, confirming the successful formation of crystalline VVLE-AgNPs (Supplementary Fig. S3).

### Growth performance at 7-day post infection

As presented in Table 1, growth performance parameters were influenced by treatments (*P* < 0.0001). Infection reduced performance in IUC broilers, as evidenced by the lowest ABW (927 g) and ADG (83.0 g). Supplementation improved growth metrics, with VVLE–AgNPs showing the most pronounced effect among challenged groups (ABW: 979 g; ADG: 93.4 g), approaching the UUC group (1000 g; 93.7 g). Feed intake (ADFI) was lowest in Tolicox (79.6 g) and highest in UUC (88.4 g), while treated groups showed moderate intake levels. FCR was significantly improved in Tolicox (0.893) and VVLE–AgNPs (0.905) compared with IUC (1.00), indicating better nutrient utilization. The performance index (PI) followed a similar pattern, with the highest value observed in VVLE–AgNPs (103), followed by Tolicox (100) and UUC (99.5), whereas IUC recorded the lowest value (83.2).

**Table 1:** Effects of experimental treatments on growth performance at 7 days post-inoculation (7 DPI) in broiler chickens challenged with *Eimeria tenella*.

Treatment^1^	IUC	Tolicox	VVLE	AgNPs	VVLE-AgNPs	UUC	SEM	*P* value
ABW d 21 (g)	927^c^	944^c^	941^c^	955^b^^c^	979^a^^b^	1000^a^	5.36	<0.0001
ADG (g)	83.0^c^	89.3^c^	88.4^c^	89.4^b^^c^	93.4^a^^b^	93.7^a^	0.719	<0.0001
ADFI (g)	82.7^b^^c^	79.6^c^	84.4^b^	84.6^b^	84.5^b^	88.4^a^	0.582	<0.0001
FCR (g/g)	1.00^a^	0.893^c^	0.955^a^^b^	0.947^a^^b^	0.905^b^^c^	0.943^b^^c^	0.008	0.0001
PI	83.2^c^	100^a^^b^	92.6^b^	94.4^b^	103^a^	99.5^a^^b^	1.46	<0.0001

1 Treatments: IUC, infected untreated control; Tolicox, infected birds treated with the standard anticoccidial drug; VVLE, infected birds treated with *Vitis vinifera* leaf extract; AgNPs, infected birds treated with biosynthesized silver nanoparticles; VVLE–AgNPs, infected birds treated with a combination of them; UUC, unchallenged untreated control. Mean data were calculated at 7 days post-infection.

a–c Letters indicate statistically significant differences (*P* < 0.05). SEM: Standard error of the mean. Abbreviations: ABW average body weight, ADG: average daily gain, ADFI: average daily feed intake, FCR: feed conversion ratio, and PI: performance index.

### Carcass traits and lymphoid organs

As presented in Table 2, carcass weight was affected by treatments (*P* = 0.003). The UUC group showed the highest carcass weight (709.3 g), while all challenged groups had lower and statistically similar values (562.7–597.0 g). Carcass yield and major cut proportions (breast and legs) were not influenced (*P* > 0.05). However, immune organ indices, particularly spleen and bursa of Fabricius were markedly affected. The spleen weight was significantly increased in the Tolicox group (0.270%) compared with other treatments (*P* < 0.0001), suggesting enhanced immune activation. Bursa weight was highest in UUC (0.433%) and lowest in infected groups, although VVLE–AgNPs partially restored bursal development (0.343%). Thymus and pancreas weights were not affected (*P* > 0.05). Edible giblets showed marginal variation (*P* = 0.05), with slightly higher values in IUC and VVLE groups

**Table 2:** Effects of experimental treatments on the carcass traits at 7 DPI in broiler chickens challenged with *Eimeria tenella*.

Treatment^1^	IUC	Tolicox	VVLE	AgNPs	VVLE-AgNPs	UUC	SEM	*P* value
Carcass weight (g)	562.7^b^	588.3^b^	563.0^b^	566.2^b^	597.0^b^	709.3^a^	13.42	0.003
Carcass yield (%)	61.8	62.6	61.7	63.3	63.4	64.7	0.365	0.138
Component weight relative to carcass weight (%)								
Breast	37	38.1	36.4	39.6	36.5	33.8	0.634	0.15
Legs	39	36.8	38.4	38.6	36.6	39.4	0.361	0.121
Spleen	0.151^b^	0.270^a^	0.140^b^	0.165^b^	0.172^b^	0.135^b^	0.01	<0.0001
Bursa	0.288^b^	0.257^b^	0.289^b^	0.325^b^	0.343^a^^b^	0.433^a^	0.014	0.001
Thymus	0.899	0.978	1.01	0.999	0.882	0.812	0.023	0.074
Pancreas	0.646	0.722	0.65	0.692	0.557	0.591	0.021	0.215
Edible giblets^2^	11.5	10.6	11.4	11.3	10.8	10.1	0.159	0.05

1 Treatments abbreviations are defined in Table 1. SEM.

2 Edible offal: Edible giblets = Liver weight + heart weight + gizzard weight.

a–c Letters indicate statistically significant differences (*P* < 0.05). SEM: Standard error of the mean.

### Clinical signs score

**Fig. 1:**
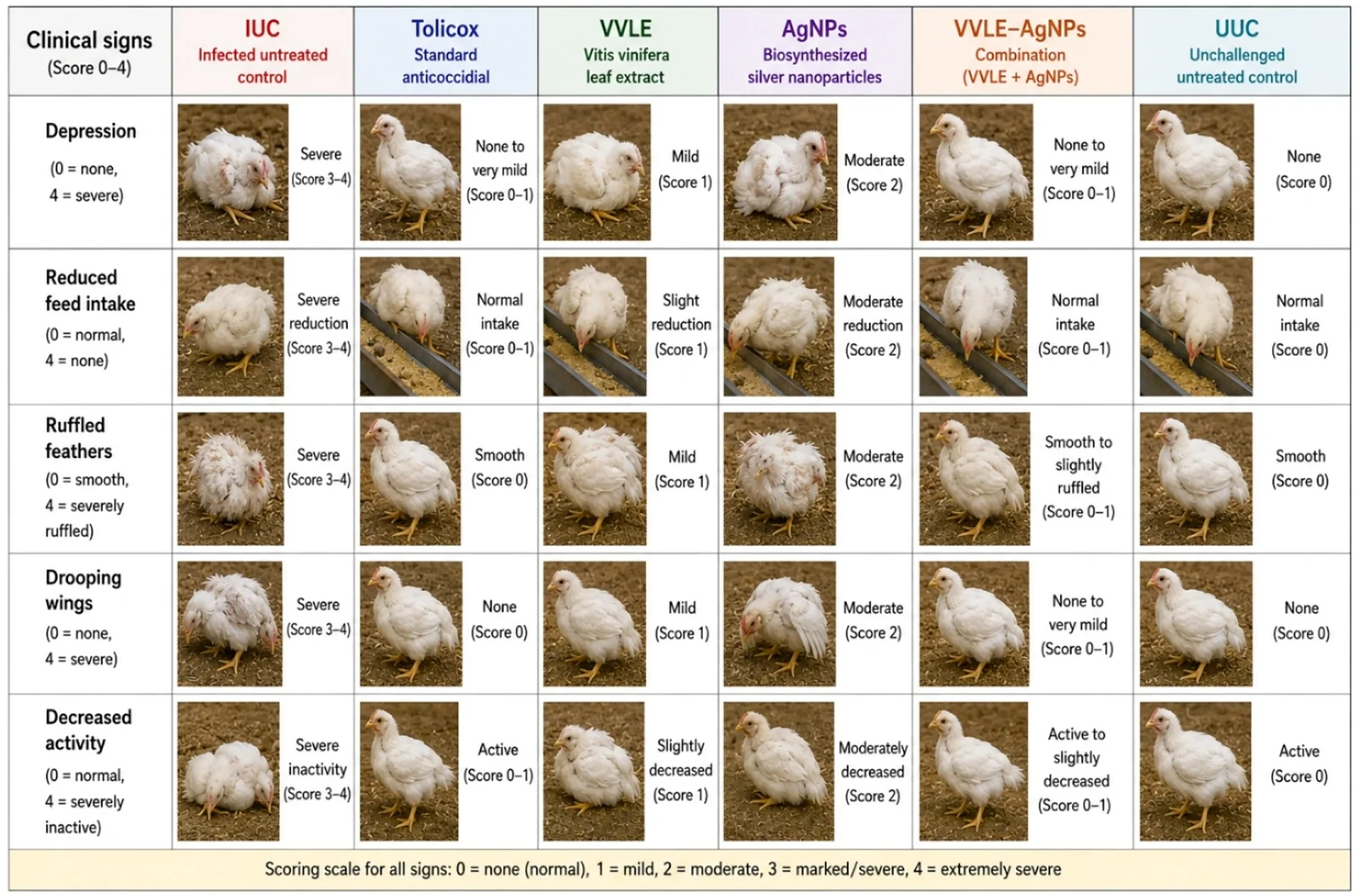
Clinical signs of coccidiosis in broilers challenged with *Eimeria tenella*. Clinical signs were scored on a 0–4 scale (0 = absent, 1 = mild, 2 = moderate, 3 = severe, and 4 = very severe). Treatment abbreviations: IUC, infected untreated control; Tolicox, infected birds treated with the reference anticoccidial drug; VVLE, infected birds treated with *Vitis vinifera* leaf extract; AgNPs, infected birds treated with biosynthesized silver nanoparticles; VVLE-AgNPs, infected birds treated with *Vitis vinifera* leaf extract-mediated biosynthesized silver nanoparticles; UUC, unchallenged untreated control.

Marked differences in the severity of clinical signs were observed among the experimental groups following the *E. tenella* challenge (Table 3 and Fig. 1). Clinical signs of coccidiosis differed markedly among treatments. Broilers in the IUC group exhibited the most severe clinical manifestations, including depression, reduced feed intake, ruffled feathers, drooping wings, and decreased activity. All treatments alleviated these symptoms to varying degrees. The lowest clinical signs scores were observed in the Tolicox and VVLE-AgNPs groups, which were comparable to those of the UUC. Birds treated with VVLE alone showed mild clinical signs, whereas those receiving AgNPs exhibited moderate symptoms. Accordingly, the severity of clinical signs followed the order: IUC > AgNPs > VVLE > Tolicox ≈ VVLE-AgNPs ≈ UUC. Among the phytogenic treatments, the VVLE-AgNPs combination was the most effective, resulting in the lowest incidence of clinical signs and exhibiting efficacy comparable to that of Tolicox. These improvements were consistent with the reductions in bloody diarrhea, lesion scores, and oocyst shedding, indicating effective mitigation of *E. tenella* infection and improved overall health status.

**Table 3:** Effects of experimental treatments on oocyst shedding, fecal bloody diarrhea, lesion score, and survival rate in *Eimeria tenella*-challenged broilers at 7 days post-infection.

Treatment^1^	IUC	Tolicox	VVLE	AgNPs	VVLE-AgNPs	UUC	SEM^2^	*P* value
Survival rate	95	100	100	100	100	100	0.833	0.439
Clinical signs score	2.67^a^	0.17^c^	0.42^bc^	0.75^b^	0.17^c^	0.00^c^	0.14	<0.0001
Bloody diarrhea score	2.58^a^	0.17^c^	0.33^c^	0.58^b^	0.17^c^	0.00^d^	0.166	<0.0001
Lesion score	2.00^a^	0.00^c^	0.11^bc^	0.44^b^	0.00^c^	0.00^c^	0.138	<0.0001
OPG output^3^	31.9^a^	2.41^c^	4.18^b^	4.80^b^	2.56^c^	0.00^d^	2.04	<0.0001

1 Treatments abbreviations are defined in Table 1.

2 SEM: Standard error of the mean.

3 Mean oocysts per gram (OPG; mean × 10^4^). Different letters (a–c) indicate statistically significant differences (*P* < 0.05).

### Anticoccidial activity and anticoccidial index at 7-day post infection

As shown in Table 3, *E. tenella* challenge markedly increased oocyst shedding, bloody diarrhea, and cecal lesion severity in the IUC. Oocyst excretion differed among treatments (*P* < 0.0001), with the highest output in IUC (31.9 × 10⁴). No oocysts were detected in the UUC, while Tolicox and VVLE–AgNPs produced the lowest shedding among challenged groups (2.41 × 10⁴ and 2.56 × 10⁴, respectively), followed by VVLE (4.18 × 10⁴) and AgNPs (4.80 × 10⁴).

Treatment also reduced clinical and pathological manifestations (*P* < 0.0001). IUC birds had the highest bloody diarrhea score (2.58) and cecal lesion score (2.00). Tolicox and the combined VVLE–AgNPs treatment completely prevented gross cecal lesions (0.00) and markedly reduced bloody diarrhea (0.17), with responses comparable to the UUC. VVLE and AgNPs alone produced intermediate reductions in bloody diarrhea and lesion severity, with lesion scores of 0.11 and 0.44, respectively.

*E. tenella* infection resulted in severe cecal lesions in the infected untreated control (lesion score = 2.00), whereas VVLE and AgNPs individually reduced lesion severity (0.11 and 0.44, respectively). Notably, the combined VVLE-AgNPs treatment completely prevented gross cecal lesions (lesion score = 0.00), comparable with the Tolicox-treated and unchallenged control groups (*P* < 0.0001). Survival rate was not affected by treatments (*P* = 0.439), remaining high (95–100%) across all groups.

**Fig. 2:**
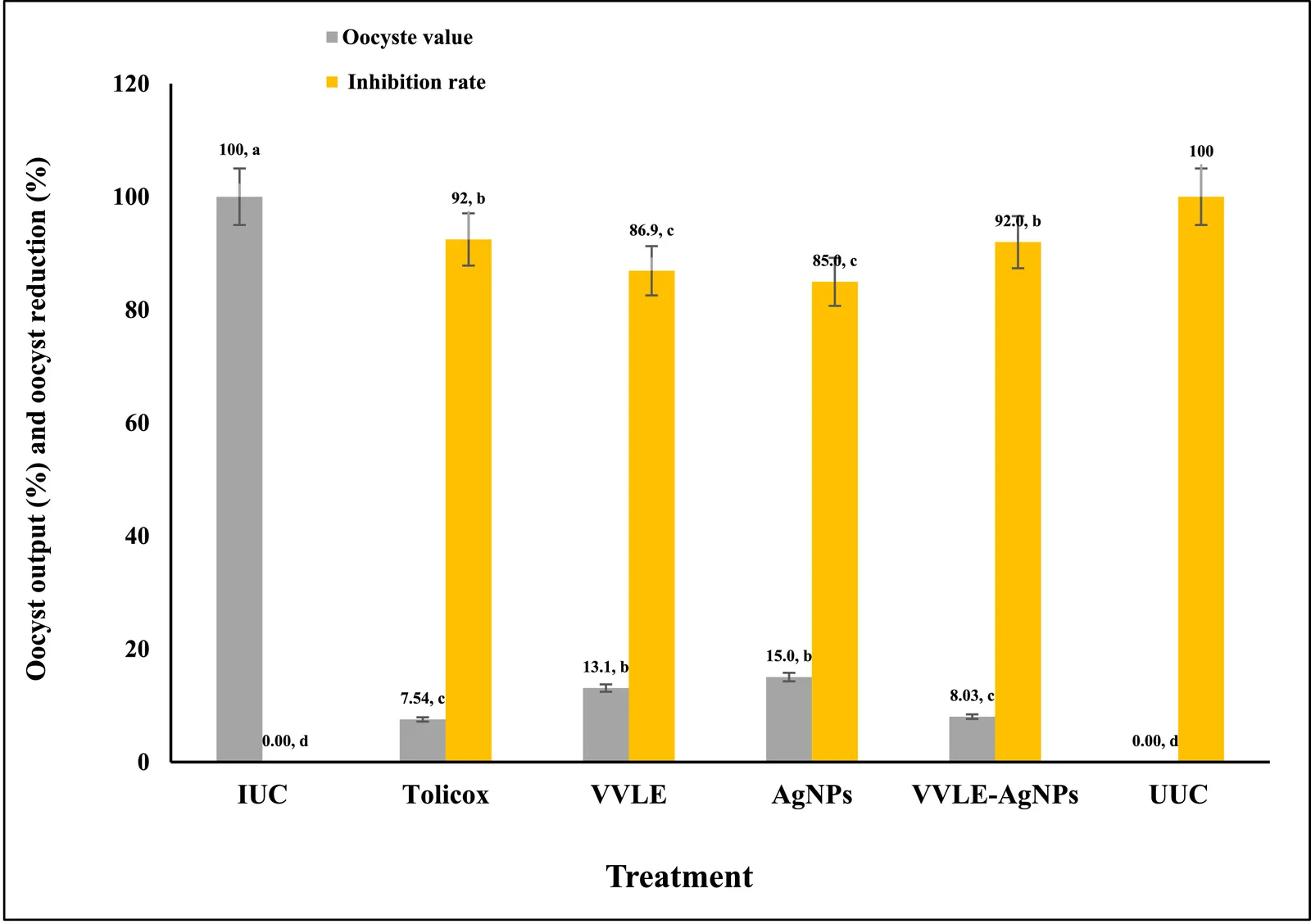
Oocyst output (%) and oocyst reduction (%) at 7 days post-infection (DPI) in broiler chickens challenged with *Eimeria tenella*. Error bars represent SEM. Different lowercase letters indicate significant differences among treatments (*P* < 0.05). Treatment abbreviations are defined in Fig. 1.

**Fig. 3:**
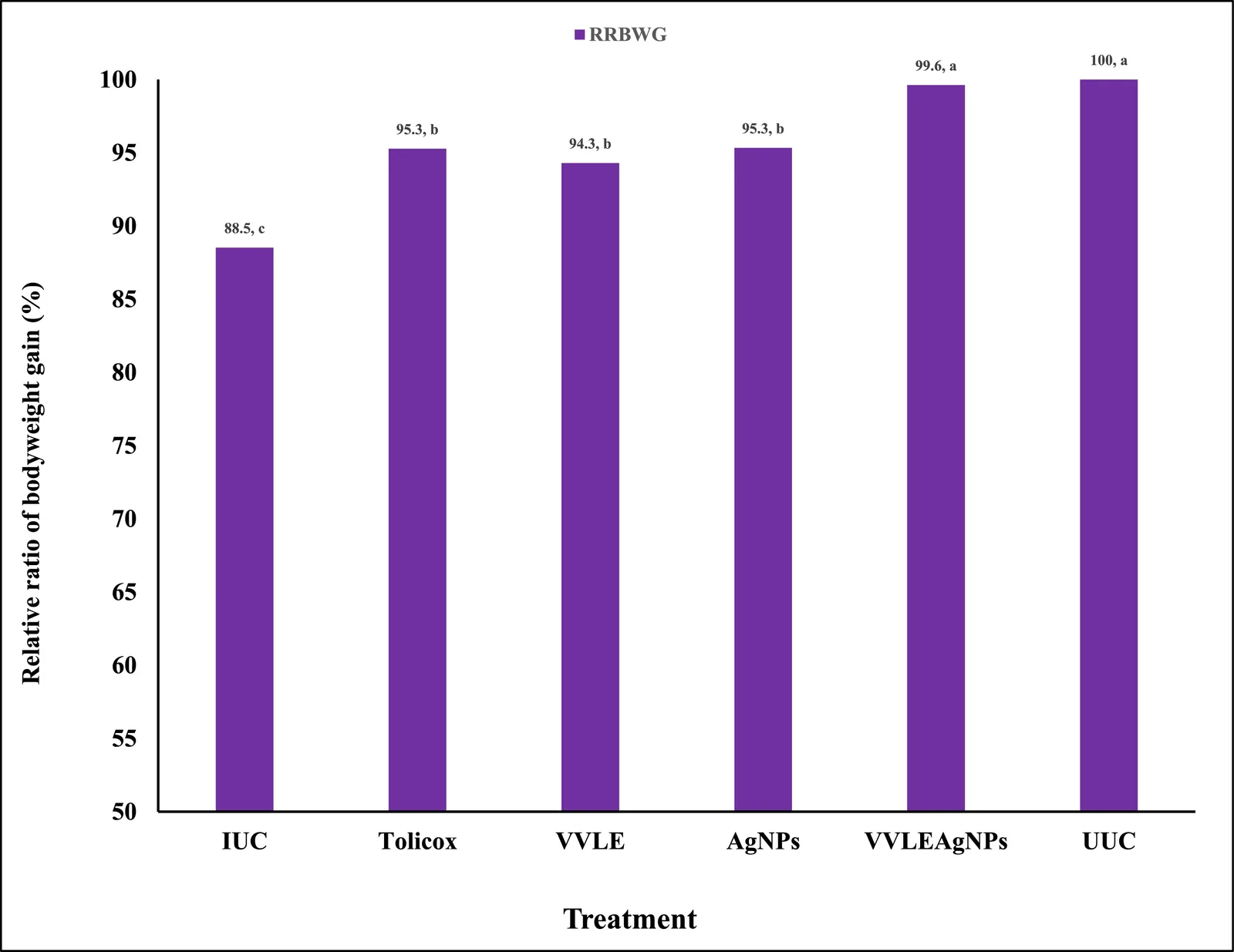
Relative body weight gain (RBWG, %) at 7 DPI in broiler chickens challenged with *Eimeria tenella*. Error bars represent SEM (0.767). Different lowercase letters indicate significant differences among treatments (*P* = 0.001). Treatment abbreviations are defined in Fig. 1.

**Fig. 4:**
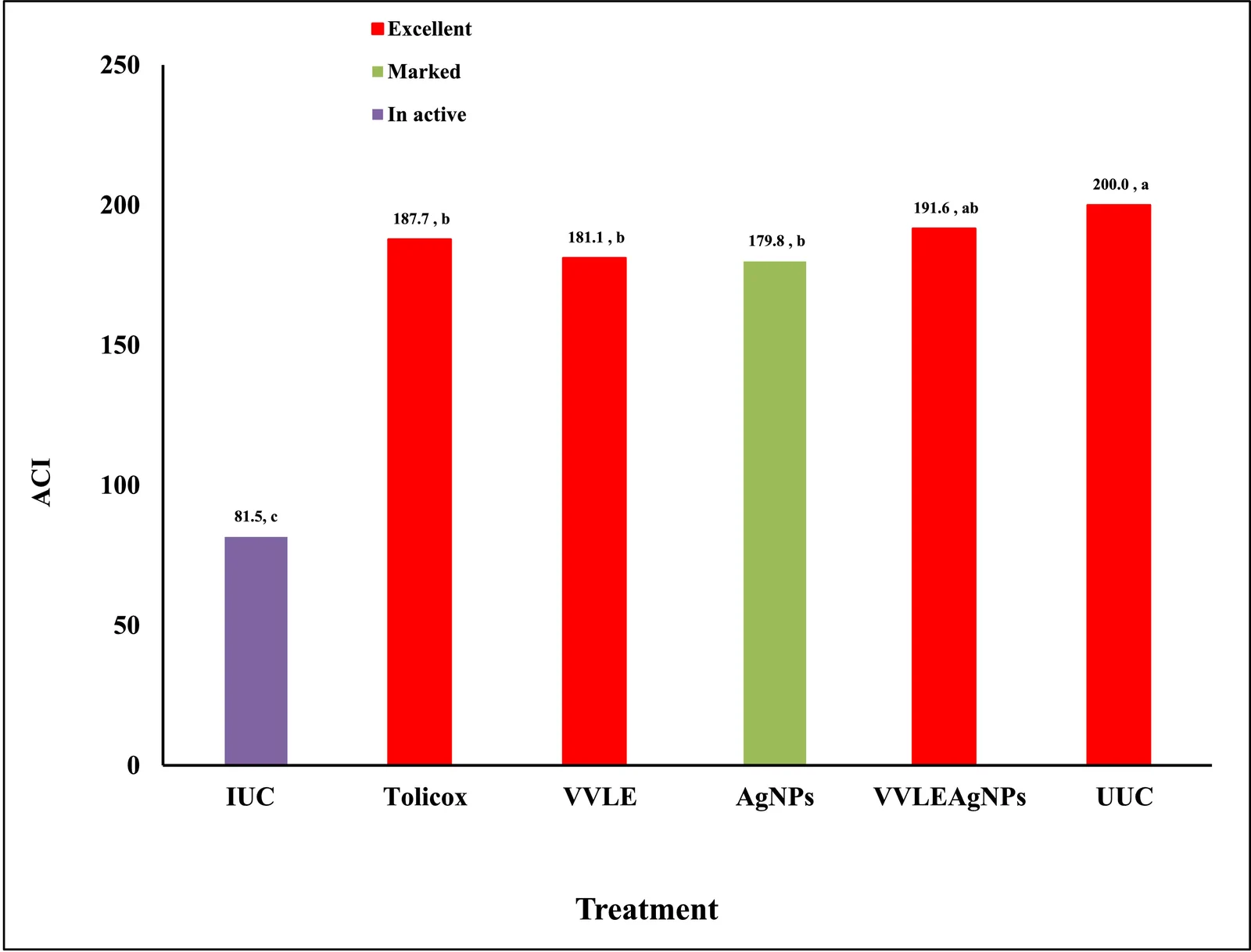
Anticoccidial index (ACI) at 7 DPI in broiler chickens challenged with *Eimeria tenella*. Different lowercase letters indicate significant differences among treatments (*P* < 0.05). Treatment abbreviations are defined in Fig. 1.

As shown in Fig. 2, the IUC exhibited the highest oocyst value (100%) with no inhibition activity (0%), confirming severe parasitic infection. In contrast, all treated groups reduced oocyst production and increased inhibition rates compared with IUC (*P* < 0.05). Among the challenged treatments, Tolicox and VVLE-AgNPs showed the greatest anticoccidial activity, recording the lowest oocyst values (7.54% and 8.03%, respectively) and the highest inhibition rates (92.0%). The VVLE and AgNPs groups showed moderate efficacy, with oocyst values of 13.1% and 15.0%, and inhibition rates of 86.9% and 85.0%, respectively. As expected, the UUC showed no detectable oocyst shedding (0%) and complete inhibition (100%). These findings indicate that the combined VVLE-AgNPs treatment effectively suppressed *E. tenella* replication and reduced oocyst excretion, with efficacy comparable to the standard anticoccidial drug. These results are supported by Fig. 3, where RBWG was significantly reduced in IUC (88.5%) and improved in all treated groups, with VVLE-AgNPs (99.6%) comparable to UUC (100%). Fig. 4 demonstrates that anticoccidial index (ACI) values clearly distinguished treatment efficacy. The UUC group recorded the highest ACI (200), while VVLE-AgNPs (191.6), Tolicox (187.7), and VVLE (181.1) were all classified as “excellent” (>180). AgNPs showed a “marked” effect (179.8), whereas IUC had a very low ACI (88.5), indicating no anticoccidial activity.

### Cecal morphometric responses and gross cecal examination appearance

**Fig. 5:**
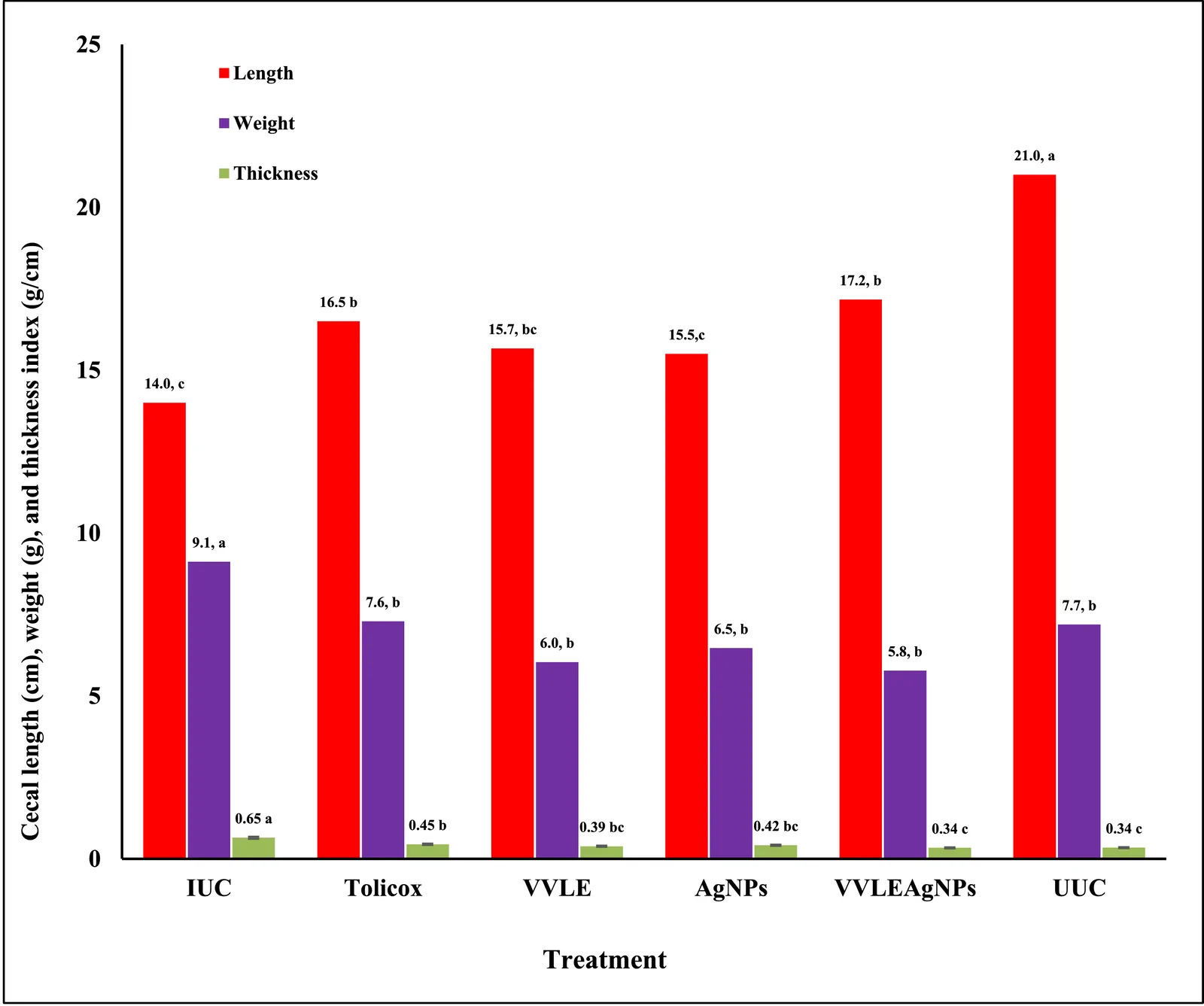
Cecal morphometric measurements (length, cm; weight, g; and thickness index, cecal weight:length ratio, g/cm) at 7 DPI in broiler chickens challenged with *Eimeria tenella*. SEM values were 0.436, 0.256, and 0.021 for cecal length, weight, and thickness index, respectively. Different lowercase letters indicate significant differences among treatments (*P* < 0.05). Treatment abbreviations are defined in Fig. 1.

Cecal morphometric responses (Fig. 5) showed that *E. tenella* infection markedly reduced cecal length while increasing cecal weight and thickness (weight: length ratio) compared with the UUC. Treatment with VVLE or AgNPs alone partially improved cecal morphometric responses, as reflected by intermediate cecal length values and significant reductions in cecal weight and thickness compared with the IUC. The combined VVLE–AgNPs treatment produced the greatest improvement among the treated groups by increasing cecal length to a level comparable with that of the Tolicox-treated group while significantly reducing cecal weight and thickness to values similar to those of the UUC (< 0.0001), indicating substantial recovery from *E. tenella*-induced cecal damage.

**Fig. 6:**
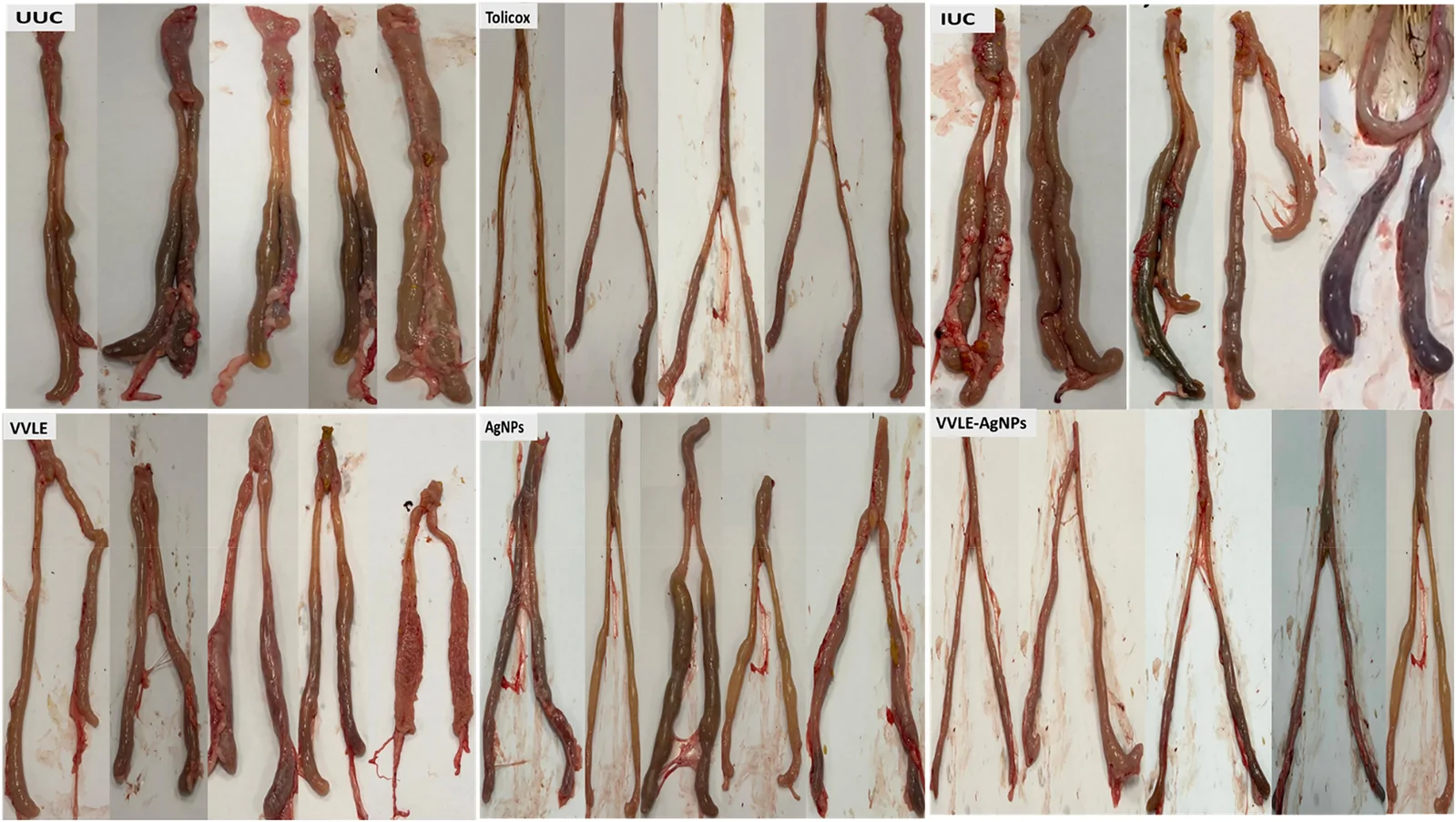
Representative gross appearance of the ceca at 7 DPI in broiler chickens challenged with *Eimeria tenella*. Treatment abbreviations are defined in Fig. 1.

Gross examination of the ceca (Fig. 6) further demonstrated severe pathological changes in the IUC group, including hemorrhage, cecal shortening, mucosal atrophy, and tissue disruption. In contrast, the treated groups, particularly the VVLE–AgNPs and Tolicox groups, exhibited marked improvement in gross cecal appearance, characterized by reduced pathological alterations and partial restoration of normal cecal morphology.

### Cecal histopathology

**Fig. 7:**
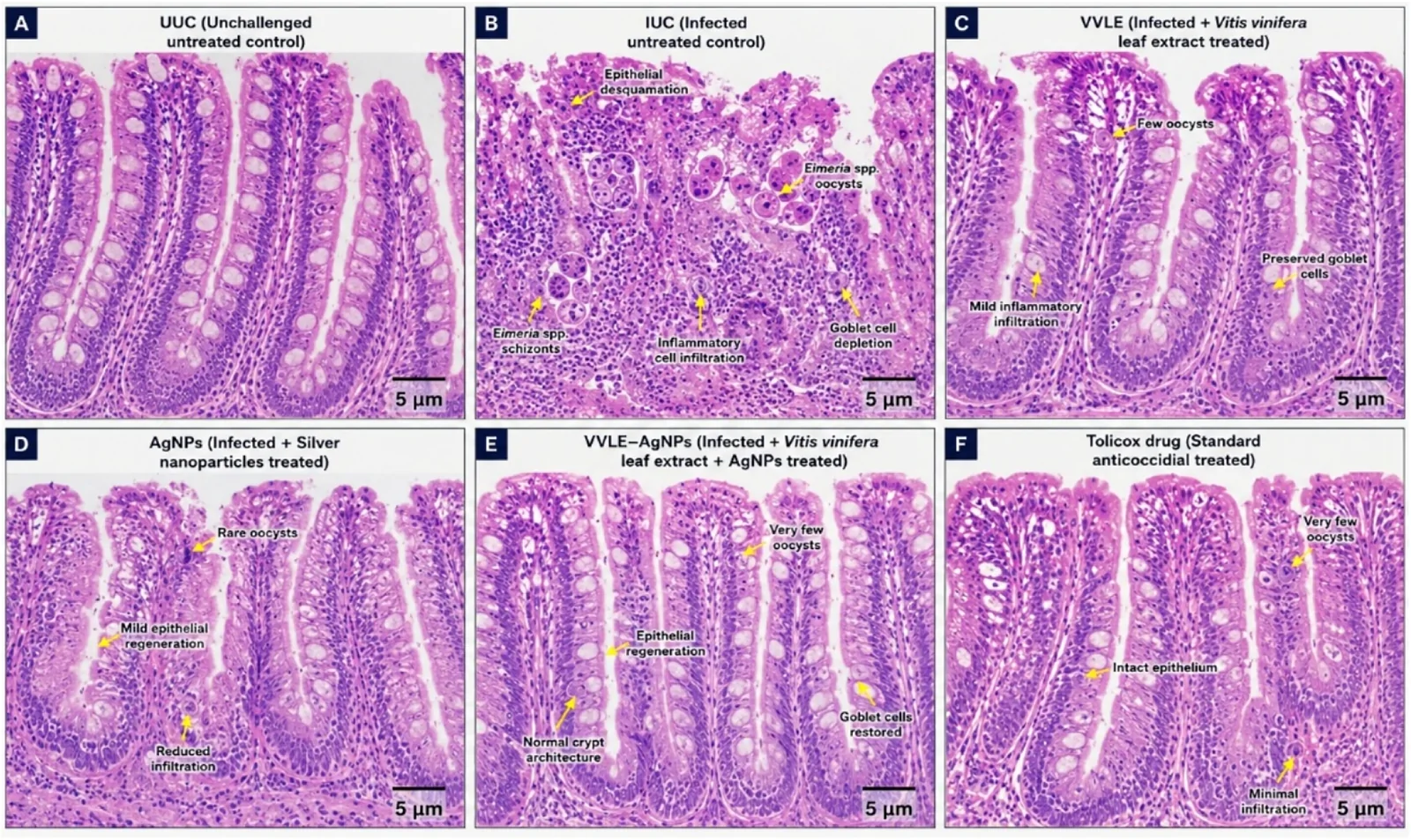
Representative hematoxylin and eosin (H&E)-stained sections of cecal tissue collected at 7 DPI from broiler chickens challenged with *Eimeria tenella*. (A) UUC, unchallenged untreated control; (B) IUC, infected untreated control; (C) VVLE, infected birds treated with *Vitis vinifera* leaf extract; (D) AgNPs, infected birds treated with biosynthesized silver nanoparticles; (E) VVLE–AgNPs, infected birds treated with *Vitis vinifera* leaf extract combined with biosynthesized silver nanoparticles; and (F) Tolicox, infected birds treated with the reference anticoccidial drug. Arrows indicate representative histopathological lesions and identifiable Eimeria developmental forms. Magnification, 100 ×; scale bar = 5 µm.

Cecal histopathology (Fig. 7) showed normal mucosal architecture with intact villi and crypt structures in the UUC (Fig. 7A). In contrast, the IUC (Fig. 7B) exhibited marked epithelial disruption and desquamation, inflammatory cell infiltration, mucosal degeneration, goblet-cell depletion, and numerous identifiable developmental forms of *E. tenella*. Treatment with VVLE (Fig. 7C) or AgNPs (Fig. 7D) partially preserved cecal architecture and reduced tissue damage compared with the IUC. The VVLE–AgNPs group (Fig. 7E) showed comparatively well-preserved epithelial and crypt architecture, minimal inflammatory changes, and fewer identifiable parasitic forms, with findings broadly comparable to those observed in the Tolicox-treated group (Fig. 7F). Representative histopathological lesions and identifiable *E. tenella* developmental forms are indicated by arrows in Fig. 7.

## Discussion

To the best of our knowledge, this is the first study evaluating VVLE-mediated AgNPs as an anticoccidial intervention against *E. tenella* in broiler chickens. The present study clearly demonstrated the detrimental impact of *E. tenella* infection on broiler performance, intestinal health, and anticoccidial indices. Birds in the IUC group exhibited lower body weight gain, poorer feed efficiency, more severe clinical signs, greater oocyst shedding, and pronounced cecal lesions compared with the other groups. These findings are consistent with the known pathogenic effects of *E. tenella*, which causes extensive destruction of the cecal epithelium, hemorrhage, impaired nutrient absorption, and diversion of nutrients toward immune responses and tissue repair rather than growth. Similar reductions in growth performance and intestinal integrity following *E. tenella* challenge have been reported previously in experimentally infected broilers (Chhetri et al., 2026; Han et al., 2022; Hassan et al., 2024; Qaid et al., 2021a; Qasem et al., 2020).

The improved ABW, ADG, FCR, and PI observed in the VVLE-AgNPs group indicate that the combined treatment effectively alleviated the adverse effects of *E. tenella* infection. Growth performance approached that of the unchallenged control, likely reflecting improved intestinal integrity and nutrient utilization associated with reduced parasite burden. Similar improvements have been reported for phytogenic anticoccidial agents that preserve intestinal function and enhance growth performance (Han et al., 2022).

The superior efficacy of VVLE-AgNPs compared with VVLE or AgNPs alone suggests that nanoparticle biosynthesis enhanced the biological activity of grapevine phytochemicals. *Vitis vinifera* leaves are rich in phenolic compounds, flavonoids, and tannins with documented antioxidant, antimicrobial, and antiparasitic properties (Murshed et al., 2024). The improved efficacy may therefore reflect enhanced stability and delivery of these bioactive compounds, although the underlying mechanisms remain to be elucidated.

Successful biosynthesis of VVLE-AgNPs was confirmed by UV-Vis spectroscopy, FTIR, TEM, EDX, and XRD analyses, which verified nanoparticle formation, morphology, elemental composition, and crystalline structure. These physicochemical characteristics are important determinants of nanoparticle stability and biological activity (El-Seedi et al., 2019; Puri et al., 2024; Radwan et al., 2024). Nevertheless, future studies should include particle size distribution, polydispersity index, zeta potential, XPS, and stability under simulated gastrointestinal conditions to further strengthen nanoparticle characterization.

The marked reductions in oocyst shedding, bloody diarrhea, and lesion scores further confirm the strong anticoccidial activity of VVLE–AgNPs. Oocyst shedding is one of the most important indicators of parasite replication and transmission potential, and the approximately 92% inhibition achieved by the combined treatment was nearly identical to that observed with the reference anticoccidial drug Tolicox. This level of suppression suggests that VVLE-AgNPs effectively interfered with the endogenous development of *E. tenella*. Comparable reductions in oocyst output have been reported for green-synthesized metallic nanoparticles used against avian coccidiosis. Khan et al. (2023) reported that iron oxide nanoparticles synthesized using *Ficus racemosa* leaf extract reduced oocyst shedding by approximately 91% while improving lesion recovery in challenged broilers. Likewise, zinc oxide and selenium nanoparticles have been shown to suppress oocyst production and improve gut health and productive performance in *Eimeria*-infected birds (Alsulami and El-Saadony, 2023; El-Maddawy et al., 2022; Lail et al., 2023).

The high ACI obtained in the VVLE-AgNPs group provides additional evidence of its efficacy. According to established classification criteria, the ACI value of this treatment was rated as excellent and was comparable to that of Tolicox. Because the ACI integrates survival rate, body weight gain, lesion severity, and oocyst production into a single parameter, it is considered one of the most comprehensive indicators of anticoccidial efficacy (Nouri, 2022). The excellent ACI observed in the present study reflects the ability of VVLE-AgNPs to simultaneously improve productive performance while reducing disease severity. Similar findings have been reported for both plant-derived anticoccidial products and nanoparticle-based interventions (Han et al., 2022; Khan et al., 2023).

Clinical observations closely supported the parasitological findings. Birds in the IUC group showed severe depression, bloody diarrhea, reduced feed consumption, ruffled feathers, drooping wings, and lethargy, all of which are characteristic manifestations of cecal coccidiosis. These signs were markedly reduced in birds treated with VVLE-AgNPs or Tolicox, indicating effective control of disease progression. These observations were consistent with the marked reductions in oocyst shedding, lesion scores, and cecal pathology. Similar improvements in clinical condition have been reported in broilers receiving phytogenic extracts and nanoparticle-based anticoccidial treatments (Han et al., 2022; Hassan et al., 2024). The significant reduction in gross lesion scores observed in the VVLE–AgNPs group was consistent with the improvements in clinical signs, bloody diarrhea, oocyst shedding, cecal morphometric responses, and histopathological findings, collectively demonstrating effective protection against *E. tenella*-induced intestinal damage.

Although carcass yield and the proportions of breast and leg muscles were not significantly affected, carcass weight remained lower in challenged birds than in the UUC. This finding suggests that the short-term infection primarily affected overall growth rather than carcass composition. Similar observations have been reported in broilers experimentally infected with *Eimeria*, where live body weight and carcass weight were reduced without substantial changes in carcass distribution (Alsulami and El-Saadony, 2023; Lail et al., 2023). The greater carcass weight observed in healthy birds is also consistent with the findings of Čobanović et al. (2024), who reported negative effects of coccidial infection on carcass development in untreated broilers

The responses of lymphoid organs provide additional insight into host immunity during infection. The reduction in bursa weight observed in infected birds is indicative of immune stress associated with coccidiosis. Interestingly, VVLE-AgNPs partially restored bursal development, suggesting partial preservation of lymphoid tissue during infection. Previous studies have shown that plant polyphenols and nanoparticle formulations can enhance immune responses through their antioxidant properties and modulation of inflammatory pathways (Conte et al., 2017; Mendonce et al., 2026; Sabzi et al., 2026). The increased spleen weight observed in the Tolicox group may reflect heightened immune activity during recovery from infection, a response also reported in broilers receiving nanoparticle-based anticoccidial treatments (Alsulami and El-Saadony, 2023; Lail et al., 2023).

Gross cecal morphometric measurements further supported the protective effects of VVLE-AgNPs against *E. tenella*-induced intestinal damage. Infection caused marked cecal shortening accompanied by increased cecal weight and thickness, reflecting hemorrhage, edema, and inflammatory swelling characteristic of acute cecal coccidiosis (Mebratie et al., 2025). Treatment with VVLE or AgNPs alone partially alleviated these pathological changes, whereas the combined VVLE-AgNPs treatment produced the greatest improvement by significantly increasing cecal length and reducing cecal weight and thickness toward values observed in the unchallenged birds. These gross anatomical findings were consistent with the histopathological observations, which demonstrated better preservation of mucosal architecture and reduced inflammatory lesions in the VVLE-AgNPs group.

Histopathological examination further corroborated the parasitological and growth performance findings. Severe epithelial disruption, inflammatory cell infiltration, hemorrhage, and numerous developmental stages of *E. tenella* were observed in the ceca of IUC birds, confirming active infection and extensive tissue damage. In contrast, the VVLE-AgNPs-treated group exhibited marked preservation of cecal architecture, reduced inflammatory infiltration and hemorrhage, and fewer parasitic developmental stages within the mucosa. These findings indicate improved mucosal integrity and attenuation of parasite-induced tissue injury, consistent with the favorable changes observed in cecal morphometric measurements. Similar histopathological improvements have been reported in *Eimeria*-challenged broilers treated with plant extracts, selenium nanoparticles, zinc oxide nanoparticles, and green-synthesized iron oxide nanoparticles (Alsulami and El-Saadony, 2023; El-Maddawy et al., 2022). Although these findings agreed with the parasitological and gross pathological results, quantitative histological scoring was not included in the original experimental design. Future studies should incorporate blinded histological scoring and morphometric analyses to provide a more objective assessment of tissue protection.

Although the present study demonstrated clear anticoccidial efficacy of VVLE-AgNPs, the underlying molecular mechanisms were not directly investigated. Previous studies have shown that grape-derived polyphenols possess antioxidant and antiparasitic activities, whereas AgNPs may disrupt parasite membranes and interfere with essential cellular processes (Mustafa et al., 2025). These mechanisms may contribute to the protective effects observed in the present study but require confirmation through analyses of inflammatory cytokines, oxidative stress biomarkers, immune-related gene expression, and intestinal barrier markers.

Overall, VVLE-AgNPs effectively controlled *E. tenella* infection, improved growth performance, reduced parasite replication, preserved cecal integrity, and achieved anticoccidial efficacy comparable with that of a conventional anticoccidial drug. These findings support the potential of green-synthesized nanoparticle-phytochemical systems as sustainable alternatives for coccidiosis control in poultry production. Nevertheless, the study was limited to the acute phase of infection under controlled experimental conditions, and the molecular mechanisms underlying the observed responses were not directly investigated. Although comprehensive nanoparticle characterization was performed using UV-Vis spectroscopy, FTIR, TEM, EDX, and XRD analyses, additional characterization (particle size distribution, polydispersity index, zeta potential, XPS, and gastrointestinal stability) would provide a more complete understanding of nanoparticle behavior. Furthermore, the experimental design was not intended to distinguish additive from synergistic effects or to compare biosynthesized with chemically synthesized AgNPs. Future studies should therefore incorporate chemically synthesized AgNP controls, factorial dose-response designs, quantitative histopathological scoring, molecular and immunological analyses, and long-term safety evaluations under commercial production conditions.

## Conclusion

The present study demonstrated that oral supplementation with VVLE, AgNPs, and particularly their combination, effectively mitigated the adverse effects of *E. tenella* infection in broiler chickens. The combined VVLE-AgNPs treatment significantly reduced oocyst shedding, bloody diarrhea, lesion severity, and cecal damage while improving growth performance, feed efficiency, anticoccidial index, and intestinal integrity. Its efficacy was comparable to that of the conventional anticoccidial drug Tolicox, supporting the potential of plant-mediated AgNPs as a sustainable alternative for coccidiosis control in poultry production.

Future studies should investigate molecular and immunological mechanisms underlying the protective effects of VVLE-AgNPs, including inflammatory cytokines, oxidative stress biomarkers, immune-related gene expression, gut barrier function, and gut microbiota. Additionally, research should also evaluate dose-optimization, long-term safety, residue profiles, comprehensive nanoparticle characterization, and efficacy under commercial production conditions to facilitate practical application.

## Data availability

The data that support the findings of this study are available on request from the corresponding author upon reasonable request.

## Funding

This study was funded by Deanship of Scientific Research and Libraries, (RG-2025-23), at Princess Nourah bint Abdulrahman University, Riyadh, Saudi Arabia.

## Author Contribution

Hind Alzaylaee conceived and designed the study, performed the experiments, analyzed and interpreted the data, and drafted the manuscript. Hend M. Alharbi and Hind Althagafi contributed substantially to the experimental work and data collection and critically revised the manuscript for important intellectual content. Wafa Abdullah I. Al-Megrin contributed substantially to data collection and critically revised the manuscript for important intellectual content. Mohammed M. Qaid, Jameel Al-Tamimi, and Saleh Al-Quraishy contributed substantially to the study through supervision, provision of research resources, interpretation of the findings, and critical revision of the manuscript. Mutee Murshed contributed substantially to the study design, supervision, methodology, interpretation of the findings, and critical revision of the manuscript. All authors reviewed and approved the exact final version of the manuscript and agree to be personally and publicly accountable for all aspects of the work, including ensuring that any questions concerning the accuracy or integrity of the research are appropriately investigated and resolved.

## Disclosures

The authors declare that they have no known competing financial or personal interests that could have appeared to influence the work reported in this paper.

## Footnotes

Supplementary material associated with this article can be found, in the online version, at doi:10.1016/j.psj.2026.107605.
