# Supplementary material for: Efficacy of Vitis vinifera leaf extract and its biosynthesized silver nanoparticles against Eimeria tenella in broilers: Effects on performance, anticoccidial indices, and cecal integrity
Source: Poult Sci. 2026 Aug 12;105(11):107605. doi: 10.1016/j.psj.2026.107605 (PMC13583756; doi:10.1016/j.psj.2026.107605)
Supplement: Supplementary file 1 [file mmc1.docx]

# **Supplementary Figures**

**Supplementary Figure S1.** UV–visible absorption spectrum confirming the biosynthesis of silver nanoparticles using Vitis vinifera leaf extract (VVLE-AgNPs).

**Supplementary Figure S2**. Physicochemical characterization of biosynthesized silver nanoparticles synthesized using *Vitis vinifera* leaf extract (VVLE-AgNPs): (A) Fourier-transform infrared (FTIR) spectrum showing the functional groups involved in nanoparticle reduction and stabilization; (B) transmission electron microscopy (TEM) image showing nanoparticle morphology and particle size; and (C) energy-dispersive X-ray spectroscopy (EDX) spectrum showing the elemental composition of the biosynthesized nanoparticles.

**Supplementary Figure S3.** X-ray diffraction (XRD) pattern confirming the crystalline structure of biosynthesized silver nanoparticles synthesized using Vitis vinifera leaf extract (VVLE-AgNPs).
