# Supplementary material for: Efficacy of Vitis vinifera leaf extract and its biosynthesized silver nanoparticles against Eimeria tenella in broilers: Effects on performance, anticoccidial indices, and cecal integrity
Source: Poult Sci. 2026 Aug 12;105(11):107605. doi: 10.1016/j.psj.2026.107605 (PMC13583756; doi:10.1016/j.psj.2026.107605)
Supplement: Supplementary file 2 [file mmc2.docx]

# **Supplementary Tables**

**Supplementary Table S1.** Diet composition and amount of feed required for broiler basal diets (%).

| **Ingredient** | **Starter (1-14 days)** | **Grower (15-21 days)** |
| --- | --- | --- |
|  | **%** | **%** |
| Corn | 53.84 | 61.43 |
| Soybean meal (48% CP) | 38.24 | 30.52 |
| Soybean oil | 3.43 | 4.40 |
| Monocalcium phosphate | 1.59 | 1.28 |
| Limestone | 1.54 | 1.28 |
| DL-Methionine | 0.37 | 0.28 |
| L-Lysine HCL | 0.25 | 0.15 |
| L-Threonine | 0.16 | 0.08 |
| Common salt | 0.38 | 0.38 |
| Vitamin premix | 0.10 | 0.10 |
| Mineral premix | 0.10 | 0.10 |
| Total | **100** | **100** |
| **Nutrient** |  |  |
| Dry matter (DM) | 88.91 | 88.92 |
| Metabolizable energy (kcal/kg) | 3000 | 3100 |
| Crude protein, % | 22.58 | 19.51 |
| Calcium, % | 0.96 | 0.79 |
| Non phytate P, % | 0.48 | 0.40 |
| D. Lysine, % | 1.28 | 1.03 |
| Sulfur amino acids, % | 0.95 | 0.80 |
| Threonine, % | 0.86 | 0.69 |
